# Type D personality, sexual functioning, sexual satisfaction, and risky sexual behaviors among Polish university students: a cross-sectional study

**DOI:** 10.3389/fpsyt.2026.1866935

**Published:** 2026-09-10

**Authors:** Iga Florczyk, Maciej Stokłosa, Gniewko Więckiewicz, Paweł Dębski, Piotr Gorczyca

**Affiliations:** Department and Clinic of Psychiatry, Faculty of Medical Sciences in Zabrze, Medical University of Silesia, Tarnowskie Góry, Poland

**Keywords:** risky sexual behaviors, sexual dysfunction, sexual functioning, sexual satisfaction, Type D personality, university students

## Abstract

**Background:**

The relationship of Type D personality with sexual functioning, satisfaction, and risky sexual behaviors in young adults remains unclear.

**Methods:**

This exploratory cross-sectional study used voluntary convenience sampling through Facebook student groups. It included 316 Polish university students reporting sexual initiation (63.0% women; age 26.2 ± 5.8 years). Type D personality was assessed with the DS14. Women completed the Female Sexual Function Index (FSFI), men completed the Index of Premature Ejaculation (IPE), and all participants completed the Sexual Risk Survey (SRS). Group comparisons used Mann–Whitney U and chi-square tests with sex-specific false discovery rate correction. Hierarchical regression examined FSFI, IPE, lifetime sexual satisfaction, and SRS after adjustment for available covariates.

**Results:**

Type D personality was identified in 49.4% of participants. In unadjusted analyses, women with Type D personality had lower FSFI total scores (p < 0.001) and higher odds of meeting the FSFI cut-off (OR = 2.26, p = 0.011). After adjustment, Type D personality remained associated with lower FSFI scores in women (β = −0.24, p = 0.001), lower lifetime sexual satisfaction in women (β = −0.18, p = 0.008) and men (β = −0.39, p < 0.001), and lower SRS total scores in the combined sample (β = −0.12, p = 0.028). It was not associated with IPE scores (β = 0.07, p = 0.486).

**Conclusions:**

Type D personality was associated with poorer female sexual functioning, lower lifetime sexual satisfaction, and lower sexual risk scores, but not with IPE-assessed outcomes. The cross-sectional, self-selected sample precludes causal inference and limits generalizability.

## 1 Introduction

Sexual health is an integral component of overall health and quality of life, encompassing physical, emotional, psychological, and social well-being rather than merely the absence of disease or dysfunction (1). University education commonly overlaps with late adolescence and young adulthood, a period characterized by ongoing neurobiological and self-regulatory maturation, delayed traditional role transitions, identity formation, increasing autonomy, and the development of close relationships (2–4). Sexuality and intimacy are therefore particularly relevant dimensions of well-being during this developmental stage.

University students are also vulnerable to mood and anxiety disorders and subclinical psychological distress, which represent a substantial public health concern (5). Sexual functioning is sensitive to mental health, chronic stress, and interpersonal context, and depressive and anxiety symptoms have been associated with difficulties in desire, arousal, orgasm, and pain in young women and men (6, 7). Clinically significant sexual dysfunction is multifactorial and requires consideration of symptoms, distress, and relational context (8, 9). Nevertheless, sexual difficulties are common in non-clinical populations: questionnaire-based studies indicate a substantial risk of sexual dysfunction among female students, while erectile and ejaculatory difficulties in young men frequently co-occur and are associated with psychological symptoms (10, 11). These findings support the investigation of psychological and personality-related correlates of sexual well-being in this age group.

Type D (distressed) personality may be relevant in this context. It is defined by the co-occurrence of negative affectivity, a stable tendency to experience distressing emotions, and social inhibition, a tendency to suppress emotional expression and feel insecure or inhibited in social interactions. Type D personality is commonly identified with the DS14 when established cut-off values are met on both dimensions (12). It has been associated with greater psychological distress, depressive and anxiety symptoms, less adaptive coping, and lower perceived social support (13). Initially studied mainly in cardiovascular populations, Type D personality has subsequently been linked to poorer quality of life and psychological or social functioning in other clinical groups, including patients with psoriasis, as well as to greater psychological burden and less favorable functioning among students (14–17).

The present study was guided by a biopsychosocial and stress-interpersonal perspective on sexual well-being. Within this framework, negative affectivity may be associated with rumination, tension, low mood, and heightened sensitivity to adverse bodily or relational experiences, whereas social inhibition may limit communication of sexual needs, openness, and use of partner support (18, 19). These characteristics may be relevant to sexual desire, arousal, orgasm, satisfaction, pain, and avoidance of sexual activity. The Dual Control Model provides additional context by conceptualizing sexual response as a balance between excitation and inhibition, although these dimensions were not measured in the present study (20). Less adaptive stress responses may also be associated with reduced desire and arousal or greater avoidance of sexual activity (21, 22). Accordingly, the selected outcomes represented three related but distinct domains: sexual functioning and dysfunction-related symptoms, subjective sexual satisfaction, and sexual behavior, including risky sexual behaviors.

Evidence directly linking Type D personality with sexual outcomes remains limited, particularly in non-clinical populations. Previous studies have reported associations with erectile dysfunction and premature-ejaculation-related symptoms in young men, risk of female sexual dysfunction in a small female sample, and sexual functioning or sexual life satisfaction in selected clinical populations (23–27). Recent dermatological studies have also examined Type D personality in relation to sexuality, although direct associations with sexual dysfunction have not been consistent (28, 29). Broader studies combining sexual functioning, sexual satisfaction, self-reported dysfunction symptoms, and risky sexual behaviors within one non-clinical student sample appear scarce, especially when women and men are analyzed separately. It therefore remains unclear whether Type D personality is associated primarily with poorer sexual functioning or with a broader sexual-life profile. Polish university students are relevant in this context because psychological distress, relationship formation, and sexual decision-making frequently overlap during young adulthood, while Polish evidence addressing these outcomes remains limited.

The aim of the present study was therefore to examine associations of Type D personality with sexual functioning and dysfunction-related outcomes, sexual satisfaction, and sexual behaviors, including risky sexual behaviors, among Polish university students. Because sexual functioning was assessed differently in women and men, it was interpreted as FSFI-based multidimensional functioning in women and IPE-based premature-ejaculation-related outcomes in men, supplemented by selected self-reported dysfunction symptoms in both groups. Given the limited evidence on sex-specific associations, these patterns were treated as exploratory. We hypothesized that participants with Type D personality would demonstrate: (H1) less favorable sexual functioning and dysfunction-related outcomes; (H2) lower sexual satisfaction; and (H3) a distinct pattern of sexual behaviors, including lower engagement in risky behaviors and differences in sexual history.

## 2 Materials and methods

### 2.1 Study framework and data source

The present study was part of a broader research initiative on health, psychological functioning, and sexual behaviors among university students in Poland. This analysis used an anonymous, self-administered cross-sectional questionnaire administered through Google Forms between March 1 and November 30, 2024. The online format facilitated geographically diverse participation and privacy when reporting sensitive information. Items required for eligibility and calculation of DS14, FSFI, IPE, and SRS scores were mandatory; thus, these measures had no item-level missing data among participants eligible for the respective analyses. A small number of responses were missing for selected matrix items concerning sexual difficulties in respondents and their partners. Partially completed but non-submitted questionnaires were not recorded. As IP addresses and other direct identifiers were not collected, duplicate submissions could not be formally screened, although all submissions were assessed for eligibility and internal consistency. Duplicate participation therefore cannot be entirely excluded.

### 2.2 Recruitment strategy and eligibility criteria

Participants were recruited by voluntary convenience sampling through Facebook student groups associated with major Polish academic centers and organized by university, field, or academic year. Administrator approval was obtained where required. A standardized post described the study aim, eligibility, voluntary and anonymous participation, and provided a survey link. Groups were selected pragmatically rather than from a formal sampling frame; universities, groups, and participants were not randomly selected, and no stratification or quotas were used. The sample should therefore not be considered representative of Polish university students. Participation was not incentivized, and electronic informed consent was required. Participants had to be at least 18 years old, be enrolled at a Polish higher education institution, and have sufficient Polish proficiency. The present analysis additionally included only respondents reporting prior sexual initiation, defined as any lifetime sexual activity and not limited to vaginal intercourse. Submitted questionnaires were screened for eligibility and internal consistency. Exclusions comprised failure to meet eligibility criteria, no prior sexual initiation, or logically contradictory key sexual-history data, such as an age at initiation exceeding current age or more partners in the previous 12 months than across the lifetime.

### 2.3 Measures and variables

A set of standardized instruments and author-developed items was used to capture psychological characteristics, sexual functioning, and behavioral patterns. The survey was administered in Polish. The language and adaptation status of each standardized questionnaire are described below.

Type D personality was assessed using the 14-item Type D Scale (DS14) developed by Denollet, in the validated Polish adaptation by Ogińska-Bulik, which measures two dimensions: Negative Affectivity (NA; 7 items) and Social Inhibition (SI; 7 items) (12, 30). Participants rated items on a 5-point scale, and established cut-off criteria (≥10 points in both subscales) were used to classify individuals as having Type D personality. In the present sample, the DS14 demonstrated high internal consistency for the total score (α = 0.907), as well as for the negative affectivity (α = 0.897) and social inhibition (α = 0.876) subscales. In gender-specific analyses, reliability remained high among women (total score: α = 0.913; negative affectivity: α = 0.894; social inhibition: α = 0.876) and men (total score: α = 0.899; negative affectivity: α = 0.904; social inhibition: α = 0.877).

Sexual functioning in women was evaluated using the Female Sexual Function Index (FSFI), a multidimensional questionnaire covering desire, arousal, lubrication, orgasm, satisfaction, and pain over the preceding four weeks. In addition to domain scores, a total score was calculated, with lower values indicating poorer functioning. A commonly applied threshold (≤26.55) was used to identify individuals at increased risk of clinically relevant sexual dysfunction (31). The validated Polish adaptation by Nowosielski et al. was used in the present study (32). FSFI-specific analyses were restricted to female participants who reported partnered sexual activity during the preceding four weeks. Women who reported no partnered sexual activity during this period were excluded from FSFI total score, domain score, and cut-off analyses to avoid misclassification related to zero scores reflecting lack of recent sexual activity rather than impaired sexual functioning. The FSFI showed excellent reliability for the total score (α = 0.904), with good to excellent reliability across the domains of desire (α = 0.869), arousal (α = 0.847), lubrication (α = 0.885), orgasm (α = 0.874), satisfaction (α = 0.882), and pain (α = 0.942).

Premature-ejaculation-related outcomes in men were assessed using the Index of Premature Ejaculation (IPE), which captures perceived ejaculatory control, sexual satisfaction, and distress related to ejaculatory function. Analyses included both domain-specific and overall scores (33). The IPE has been translated into several languages, including Polish. A Polish-language version was used in the present study. However, no separate peer-reviewed psychometric validation study of the Polish-language version was identified; consequently, IPE-based findings were interpreted cautiously as exploratory premature-ejaculation-related outcomes. Among men, the IPE demonstrated excellent internal consistency for the total score (α = 0.908), as well as for the satisfaction (α = 0.906), control (α = 0.906), and distress/frustration (α = 0.950) domains.

Risky sexual behaviors were measured using the Polish adaptation of the Sexual Risk Survey (SRS) by Aleksandra Barabasz-Gembczyk, which assesses the frequency of behaviors such as impulsive sexual activity, engagement with casual partners, and other forms of risk-taking in the past six months. Both total and subscale scores were analyzed (34, 35). The SRS demonstrated high internal consistency for the total score in the total sample (α = 0.906), as well as in women (α = 0.889) and men (α = 0.928). In the total sample, reliability across the SRS subscales ranged from acceptable to excellent: F1 α = 0.927, F2 α = 0.706, F3 α = 0.803, F4 α = 0.789, and F5 α = 0.785. In gender-specific analyses, Cronbach’s alpha values for F1–F5 were α = 0.912, 0.764, 0.737, 0.740, and 0.795 among women, and α = 0.935, 0.656, 0.856, 0.809, and 0.775 among men, respectively.

In addition to standardized tools, participants completed an author-developed structured questionnaire, originally created in Polish for the broader research project. The questionnaire was not adapted from a single previously validated instrument, but its content was based on the study objectives, previous literature on sexual health in young adults, and variables commonly used in survey-based studies of sexual behavior. It included sociodemographic and educational characteristics, relationship status and partner context, sexual orientation, history and age of initiation of selected sexual activities, frequency of sexual activity, number of sexual partners, subjective sexual satisfaction, and self-reported sexual difficulties in respondents and their partners. Lifetime sexual satisfaction and sexual satisfaction during the previous 12 months were each assessed using a single item on a 5-point Likert scale, with higher scores indicating greater satisfaction. Participants were also asked whether they had previously been diagnosed with depression or an anxiety disorder. These variables reflected self-reported diagnostic history and did not assess the presence or severity of current depressive or anxiety symptoms. The analyses were based on the self-reported gender category declared by participants in the author-developed questionnaire. Biological sex assigned at birth was not assessed as a separate variable, and gender identity was not assessed in detail. Therefore, the terms women and men used throughout the manuscript refer to the self-reported gender categories used for gender-specific analyses. Before online dissemination, the questionnaire was reviewed by an expert in the field of sexology for content relevance, clarity, neutral wording, cultural appropriateness, and acceptability of items addressing sensitive sexual health topics. The questionnaire was then pre-tested in a convenience sample of 30 medical university students. The pre-test assessed item comprehensibility, acceptability, completion flow, technical functionality, and branching logic. The items were considered understandable and acceptable, but they were not formally psychometrically validated and should not be treated as a separate validated instrument.

### 2.4 Ethical considerations

The study adhered to the principles outlined in the Declaration of Helsinki. The study protocol was submitted to and reviewed by the Bioethics Committee of the Medical University of Silesia in Katowice (reference no. PCN/CBN/0052/KB/214/23). The Committee confirmed that, because the study was anonymous, survey-based, and non-interventional, formal ethical approval was not required; no formal approval was issued.

Before accessing the questionnaire, all participants received information about the study aims, voluntary participation, anonymity, and the right to withdraw before submitting the form. Participants provided electronic informed consent before completing the survey. No identifying information was collected.

### 2.5 Statistical analysis

All statistical analyses were conducted using Statistica 13.3 software (StatSoft, Kraków, Poland). Descriptive statistics were used to summarize the data, with continuous variables presented as means with standard deviations or medians with interquartile ranges, as appropriate, and categorical variables expressed as frequencies and percentages. No missing values were imputed. Analyses of the author-developed questionnaire items were conducted using the valid responses available for each individual item. Consequently, item-specific denominators could vary slightly, as indicated in the relevant table notes. Regression analyses were based on complete cases for the variables included in each model. The distribution of continuous variables was assessed using the Shapiro–Wilk test. Due to deviations from normality, nonparametric methods were used for the unadjusted group comparisons. Group comparisons were performed using the Mann–Whitney U test, and the magnitude of group differences was quantified using the effect size r, calculated as |Z|/√N and interpreted as small (≥ 0.1), medium (≥ 0.3), or large (≥ 0.5). Eta-squared (η² = r²) with 95% confidence intervals was additionally reported in the tables. Differences in categorical variables were analyzed using the chi-square test or Fisher’s exact test when expected cell counts fell below 5. For 2 × 2 contingency tables, the magnitude of association was quantified using the phi coefficient (φ), and odds ratios (ORs) with corresponding 95% confidence intervals (95% CIs) were additionally reported. Exploratory hierarchical linear regression analyses using the enter method were conducted for the primary continuous outcomes. Separate sex-specific models were used to predict the FSFI total score among women, the IPE total score among men, and lifetime sexual satisfaction among women and men. A further model was conducted for the SRS total score in the combined sample. In the sex-specific models, age, stable relationship status, self-reported history of depression, self-reported history of anxiety disorder, and having children were entered in Step 1. Type D personality was added in Step 2 as the sole additional predictor to assess its incremental contribution to the explained variance. In the SRS model, Step 1 included sex, age, lifetime number of sexual partners, lifetime sexual satisfaction, having children, frequency of sexual intercourse, and self-reported history of depression and anxiety disorder; Type D personality was added in Step 2. Regression assumptions were evaluated using residual diagnostics, assessment of homoscedasticity, variance inflation factors, and Cook’s distance. Because lifetime sexual satisfaction was assessed on a bounded 5-point scale and residual diagnostics indicated heteroscedasticity in the female model, HC3 robust inference was used for both sex-specific satisfaction models to provide consistent and heteroscedasticity-robust estimates of uncertainty. Unstandardized regression coefficients with 95% confidence intervals, standard errors, standardized coefficients, p-values, adjusted R², ΔR², and ΔF are reported. Full regression results are provided in **Supplementary Table 1–S5**. Internal consistency reliability of the standardized instruments and their subscales was assessed using Cronbach’s alpha. To account for multiple testing, the Benjamini–Hochberg procedure was applied separately to two sex-specific correction families. The female correction family comprised all primary Type D versus control group comparisons and categorical cross-tabulations conducted within the female subcohort, whereas the male correction family comprised all primary Type D versus control group comparisons conducted within the male subcohort. Corrections were therefore performed separately for women and men rather than across all comparisons in the full sample. Statistical significance was set at p < 0.05. Raw, unadjusted p-values are reported in all tables, while the Benjamini–Hochberg procedure was used to determine which findings retained statistical significance after correction for multiple comparisons. Findings that did not retain statistical significance after the relevant sex-specific correction are explicitly identified in the Results section and relevant table notes. All analyzed outcomes are presented in the tables, regardless of statistical significance. Because no formal *a priori* sample size calculation was performed, a *post-hoc* statistical power analysis was conducted using G*Power software to evaluate the sensitivity of the analyses within the male subsample (N = 117). For continuous comparisons using the Mann–Whitney U test, assuming a medium effect size (d = 0.50) and a significance level of α = 0.05, the achieved statistical power was 0.76, which is slightly below the conventional 0.80 threshold and suggests somewhat limited power to detect medium-sized differences. For chi-square tests of independence (df = 1), the power to detect a medium effect size (w = 0.30) exceeded 0.90, whereas it decreased to 0.58 for small-to-medium effect sizes (w = 0.20).

## 3 Results

Participant flow was determined from submitted questionnaires only. Of 376 responses, 12 were excluded because of internally inconsistent key sexual-history data, including an age of sexual initiation exceeding current age or more partners in the previous 12 months than across the lifetime. Among the remaining 364 respondents, 48 reported no prior sexual initiation and were excluded on eligibility grounds. The final analytic sample therefore comprised 316 participants.

### 3.1 Characteristics of the study cohort

In the analyzed population, 49.4% of respondents met the criteria for Type D personality. Women constituted 63.0% of the cohort. No significant gender differences were observed in the prevalence of Type D personality. The gender distribution for this variable is presented in **Table 1**, while the general characteristics of the cohort are shown in **Table 2**. The mean age of the analyzed sample was 26.2 ± 5.8 years, with an age range of 19–50 years. Although the sample included some older students, its mean age was consistent with the young-adult focus of the study.

**Table 1:** Distribution of type D personality in the studied population.

Charateristic	All	Male	Female
	316	117	199
	100%	37.0%	63.0%
Type D personality	49.4%	44.4%	52.3%
Control group	50.6%	55.6%	47.7%

**Table 2:** Characteristics of the study population.

Charateristic	Category	All	Type D personality	Control group
N		316	156	160
%		100%	49.4%	50.6%
Age, years; mean ± SD; range		26.2 ± 5.8; 19-50	25.3 ± 5.5; 19-49	27.0 ± 6.0; 19-50
Field of study:	Artistic/Natural sciences/Sport	11.4%	10.3%	12.5%
	Economics and management	9.5%	9.6%	9.4%
	Humanities / Pedagogy / Law / Religion	15.8%	16.0%	15.6%
	Medical	32.0%	30.1%	33.8%
	Polytechnic / IT / Industry	19.0%	21.2%	16.9%
	Other	12.3%	12.8%	11.9%
Place of birth: (inhabitants)	village	24.1%	24.4%	23.8%
	<20.000	13.6%	12.2%	15.0%
	20-50.000	12.7%	14.1%	11.3%
	50-100.000	13.9%	14.7%	13.1%
	100-200.000	15.8%	12.8%	18.8%
	>200.000	19.9%	21.8%	18.1%
Place of residence:	village	13.6%	14.7%	12.5%
	<20.000	5.1%	5.1%	5.0%
	20-50.000	5.7%	5.8%	5.6%
	50-100.000	9.8%	8.3%	11.3%
	100-200.000	22.8%	20.5%	25.0%
	>200.000	43.0%	45.5%	40.6%
Where do you currently live?	In an apartment with a partner	31.6%	28.8%	34.4%
	In an apartment with friends	15.5%	13.5%	17.5%
	In a dorm	8.2%	9.6%	6.9%
	Alone	16.8%	16.0%	17.5%
	With parents	27.8%	32.1%	23.8%
Having children	No	88.0%	92.9%	83.1%
	Yes	12.0%	7.1%	16.9%
Do you live according to the principles of your religion?	No	76.9%	74.4%	79.4%
	Yes	23.1%	25.6%	20.6%

SD – standard deviation.

Statistical analysis indicated that age was significantly lower in the Type D group compared to the non–Type D group (Mann–Whitney U test; Z = 3.09; effect size r = 0.17; p = 0.002). Respondents in the control group had higher odds of reporting having children than those with Type D personality (χ² = 7.20, p = 0.007; OR = 2.68, 95% CI [1.27, 5.64]). No significant differences were observed with respect to the level of religious observance, size of hometown, or current place of residence.

### 3.2 Analysis of female respondents

#### 3.2.1 Type D personality and partner relationships in the female population

Sexual relationship characteristics of the female cohort are presented in **Table 3**. Women with Type D personality reported a significantly lower lifetime number of sexual partners (Mann–Whitney U test; Z = -2.38; p = 0.017; effect size r = 0.17). However, no significant differences were observed between the groups in the number of sexual partners within the past 12 months. Regarding marital status, respondents with Type D personality were nominally less likely to be married compared to those in the control group (χ² = 4.4; p = 0.036; OR = 2.82, 95% CI: 1.03-7.72); however, this association did not remain statistically significant after the Benjamini–Hochberg correction. No significant differences were observed between the groups with respect to being in a stable relationship, having a steady partner, or the frequency of sexual intercourse.

**Table 3:** Partner relationships in the studied group of women. N = 199.

A. Relationship and sexual activity characteristics						
Charateristic	Response category	All	Type D personality		Control group	
Sexual orientation	Asexual	1.00%	2.00%		0.00%	
	Bisexual	16.60%	16.30%		16.80%	
	Heterosexual	78.40%	77.90%		78.90%	
	Homosexual	2.00%	1.90%		2.10%	
	Other	2.00%	1.90%		2.20%	
Do you have a regular sexual partner?	Yes (one)	81.40%	83.70%		78.90%	
	Yes (many)	1.50%	0.00%		3.20%	
	No	17.10%	16.30%		17.90%	
Are you in a regular relationship:	Yes, married	10.10%	5.80%		14.70%	
	Yes, partner	44.70%	46.20%		43.20%	
	No	45.20%	48.10%		42.10%	
How often do you have sex?	<1x/month	16.80%	19.20%		14.30%	
	1-2x/month	22.60%	24.20%		20.90%	
	1-2x/week	37.40%	41.40%		33.00%	
	3-4x/week	14.20%	7.10%		22.00%	
	> 4x/week	8.90%	8.10%		9.90%	
B. Group comparisons for sexual activity and satisfaction variables						
Variable	Type D Mdn (IQR)	Control group Mdn (IQR)	Z	p	r	η² [95% CI]
Frequency of sexual intercourse	3.00 (1.00)	3.00 (2.00)	-1.9	0.057	0.13	0.02 [0.00, 0.07]
Number of sexual partners (lifetime)	2.00 (3.00)	2.00 (4.00)	-2.38	0.017	0.17	0.03 [0.00, 0.09]
Number of sexual partners (last 12 months)	1.00 (0.00)	1.00 (0.00)	-0.34	0.735	0.02	<0.01 [0.00, 0.03]
Sexual satisfaction (lifetime) (LS)	4.00 (1.00)	4.00 (1.00)	-3.07	0.002	0.22	0.05 [0.01, 0.12]
Sexual satisfaction (last 12 months) (LS)	4.00 (2.00)	4.00 (2.00)	-1.75	0.081	0.12	0.02 [0.00, 0.07]

Mdn = median; IQR = interquartile range; Z = standardized Mann–Whitney U test statistic; p = raw, unadjusted p-value; r = effect size calculated as |Z|/√N; η² = eta-squared effect size; CI = confidence interval; LS = Likert scale. The frequency-of-intercourse variable was available for 190 women; percentages and the corresponding group comparison were based on valid responses.

#### 3.2.2 Type D personality and sexual life satisfaction in the female population

Lifetime sexual satisfaction, assessed using a 5-point Likert scale, was significantly lower among respondents with Type D personality (Mann–Whitney U test; Z = -3.07; p = 0.002; effect size r = 0.22). In contrast, no significant differences were observed between the groups in sexual satisfaction over the past 12 months.

#### 3.2.3 Type D personality, specific sexual activities, and age of initiation in the female population

A comparison of engagement in specific sexual activities between the study groups is presented in **Table 4**. No significant differences were observed between the groups in the prevalence of various sexual activities, including masturbation, petting, oral sex, vaginal sex, and anal sex. Similarly, no significant differences were observed in the age of initiation of any of these activities.

**Table 4:** Sexual initiation and age at initiation of selected sexual activities in women (N = 199).

A. History of sexual initiation by type of activity						
Activity	Response	All	Type D personality			Control group
Masturbation	No	8.0%	8.7%			7.4%
	Yes	92.0%	91.3%			92.6%
Petting	No	3.5%	2.9%			4.2%
	Yes	96.5%	97.1%			95.8%
Oral sex	No	9.0%	9.6%			8.4%
	Yes	91.0%	90.4%			91.6%
Vaginal sex	No	13.1%	16.3%			9.5%
	Yes	86.9%	83.7%			90.5%
Anal sex	No	55.8%	51.0%			61.1%
	Yes	44.2%	49.0%			38.9%
B. Age at initiation by type of activity						
Activity	Type D personality Mdn (IQR)	Control group Mdn (IQR)	Z	p	r	η² [95% CI]
Masturbation	15.00 (4.00)	14.00 (3.25)	-0.32	0.747	0.02	<0.01 [0.00, 0.03]
Petting	17.00 (3.00)	17.00 (3.00)	-0.36	0.717	0.03	<0.01 [0.00, 0.03]
Oral sex	18.00 (3.00)	18.00 (2.00)	-0.67	0.504	0.05	<0.01 [0.00, 0.04]
Vaginal sex	18.00 (4.00)	18.00 (2.00)	-0.23	0.819	0.02	<0.01 [0.00, 0.03]
Anal sex	20.00 (4.00)	20.50 (4.50)	-1.29	0.198	0.14	0.02 [0.00, 0.11]

Reported p-values are raw, unadjusted p-values. Mdn – median; IQR – interquartile range; Z – standardized Mann–Whitney U test statistic; p – p-value; r = effect size calculated as |Z|/√N; η² – eta-squared effect size; CI – confidence interval.

#### 3.2.4 Type D personality and self-reported sexual dysfunction symptoms in the female population

Symptoms of sexual dysfunction in the female cohort are presented in **Table 5**. Regarding self-reported symptoms, women with Type D personality were nominally more likely to report greater difficulty achieving orgasm during masturbation compared to the control group (χ² = 4.29; p = 0.038; OR = 0.49, 95% CI [0.25, 0.97]); however, this comparison did not remain statistically significant after the Benjamini–Hochberg correction. In contrast, the higher prevalence of subjectively reported pain during intercourse among female respondents with Type D personality remained statistically significant after the Benjamini–Hochberg correction (χ² = 4.81; p =0.028; OR = 0.52, 95% CI. [0.29, 0.94]). No significant between-group differences were observed in sexual dysfunction symptoms reported by respondents in their partners.

**Table 5:** Symptoms of sexual dysfunction in the studied women (N = 199).

Question	Sexual difficulty	Response	Control group	Type D personality	χ²	p	φ	OR [95% CI]
Have you noticed any of the following sexual difficulties during the past 12 months?	Decreased sexual desire	Yes	39 (41.5)	44 (42.3)	0.01	0.907	0.01	0.97 [0.55, 1.70]
		No	55 (58.5)	60 (57.7)				
	Lubrication difficulties	Yes	28 (30.1)	33 (31.7)	0.06	0.806	0.02	0.93 [0.51, 1.70]
		No	65 (69.9)	71 (68.3)				
	Dyspareunia	Yes	33 (36.7)	53 (52.5)	4.81	0.028	0.16	0.52 [0.29, 0.94]
		No	57 (63.3)	48 (47.5)				
	Orgasmic disorder	Yes	45 (48.9)	58 (56.9)	1.23	0.268	0.08	0.73 [0.41, 1.28]
		No	47 (51.1)	44 (43.1)				
	Masturbatory anorgasmia	Yes	16 (17.4)	31 (30.1)	4.29	0.038*	0.15	0.49 [0.25, 0.97]
		No	76 (82.6)	72 (69.9)				
During the past 12 months, have you noticed any of the following sexual difficulties in your partner?	Decreased sexual desire	Yes	20 (22.2)	22 (22.4)	0.00	0.970	0.00	0.99 [0.50, 1.96]
		No	70 (77.8)	76 (77.6)				
	Erectile dysfunction	Yes	2 (2.4)	3 (3.5)	–	0.523†	0.03	0.69 [0.11, 4.25]
		No	80 (97.6)	83 (96.5)				
	Dyspareunia	Yes	6 (7.0)	5 (5.2)	0.25	0.617	0.04	1.36 [0.40, 4.64]
		No	80 (93.0)	91 (94.8)				
	Premature ejaculation	Yes	19 (21.3)	26 (26.3)	0.62	0.430	0.06	0.76 [0.39, 1.50]
		No	70 (78.7)	73 (73.7)				
	Orgasmic disorder	Yes	12 (13.6)	21 (21.6)	2.02	0.155	0.11	0.57 [0.26, 1.24]
		No	76 (86.4)	76 (78.4)				

Percentages were calculated using the number of valid responses for each item; denominators therefore vary across questions. Data are presented as n (%). χ² = chi-square test statistic; p = raw, unadjusted p-value; φ = phi coefficient (effect size); OR = odds ratio; CI = confidence interval. ORs compare the control group with the Type D personality group for the “Yes” response. * This comparison was not statistically significant after the Benjamini–Hochberg correction.

† Fisher’s exact test was used because more than 20% of the expected cell counts were < 5.

#### 3.2.5 FSFI score and domain-specific results in women with Type D personality

**Table 6** presents a comparison of the Female Sexual Function Index (FSFI) total and domain scores. FSFI analyses included 190 women who reported partnered sexual activity during the preceding four weeks; nine women who reported no partnered sexual activity during this period were excluded. The total FSFI score was significantly lower among respondents with Type D personality (Mann–Whitney U test; Z = -3.87; p < 0.001). Women with Type D personality also scored significantly lower in the domains of arousal, lubrication, orgasm, and pain. Although the lower satisfaction-domain score was nominally significant at the unadjusted level (p = 0.041), it did not remain statistically significant after the sex-specific Benjamini–Hochberg correction. No statistically significant difference was observed in the desire domain.

**Table 6:** Comparison of FSFI total and domain scores between women with Type D personality and the control group (N = 190).

Variable	Control group (n = 91) Mdn (IQR)	Type D personality (n = 99) Mdn (IQR)	Z	p	r	η² [95% CI]
FSFI total score	29.80 (6.60)	27.40 (6.60)	-3.87	<0.001	0.28	0.08 [0.02, 0.18]
D1 - desire	4.80 (1.80)	4.20 (1.20)	-1.70	0.089	0.12	0.02 [0.00, 0.07]
D2 - arousal	5.10 (1.20)	4.80 (1.50)	-2.80	0.005	0.20	0.04 [0.00, 0.12]
D3 - lubrication	5.70 (0.90)	5.40 (1.80)	-2.99	0.003	0.22	0.05 [0.01, 0.13]
D4 - orgasm	4.80 (2.00)	4.00 (1.80)	-3.00	0.003	0.22	0.05 [0.01, 0.13]
D5 - satisfaction	5.20 (1.60)	4.80 (1.60)	-2.04	0.041*	0.15	0.02 [0.00, 0.08]
D6 - pain	4.80 (1.60)	4.80 (2.20)	-2.41	0.016	0.17	0.03 [0.00, 0.10]

Data are presented as median (IQR). The groups were compared using a two-sided Mann–Whitney U test. Z = standardized Mann–Whitney U test statistic; p = raw, unadjusted p-value; r = effect size calculated as |Z|/√N; η² = eta-squared effect size; CI = confidence interval. * This comparison was not statistically significant after the Benjamini–Hochberg correction. .

#### 3.2.6 Type D personality and the FSFI cut-off for risk of clinically significant sexual dysfunction in the female population

An FSFI cut-off score of ≤26.55 was used to identify individuals at risk of clinically significant sexual dysfunction, as presented in **Table 7**. Respondents with Type D personality were significantly more likely to meet the criterion for risk of clinically significant sexual dysfunction (χ² = 6.53; p = 0.011; OR = 2.26, 95% CI [1.20, 4.25]).

**Table 7:** Comparison of groups according to the FSFI cutoff score (≤26.55) indicating risk of clinically significant sexual dysfunction (N = 190).

Risk of clinically significant sexual dysfunction	Control group (n = 91), n (%)	Type D personality (n = 99), n (%)	χ²(1)	p	φ	OR [95% CI]
No	70 (76.9)	59 (59.6)	6.53	0.011	0.185	2.26 [1.20, 4.25]
Yes	21 (23.1)	40 (40.4)				

Data are presented as n (%). χ² = chi-square test statistic; p = raw, unadjusted p-value; φ = phi coefficient (effect size); OR = odds ratio; CI = confidence interval. The OR compares the Type D personality group with the control group for meeting the FSFI cutoff criterion (“Yes”).

#### 3.2.7 Risky sexual behaviors measured by the SRS in the female population

Women with Type D personality had significantly lower SRS total scores (Z = −2.11; p = 0.035; r = 0.15) and significantly lower scores for sexual behaviors with casual partners (Z = −2.52; p = 0.012; r = 0.18). Detailed SRS results for the female population are presented in **Table 8**.

**Table 8:** Comparison of sexual risk survey scores between women with Type D personality and the control group (N = 199).

Variable	Control group (n = 95), Mdn (IQR)	Type D personality (n = 104), Mdn (IQR)	Z	p	r	η² [95% CI]
SRS total score	21.00 (19.00)	16.00 (17.75)	-2.11	0.035	0.15	0.02 [0.00, 0.08]
F1 - sexual behaviors with casual partners	5.00 (9.00)	2.00 (6.75)	-2.52	0.012	0.18	0.03 [0.00, 0.10]
F2 - risky sexual intercourse	11.00 (9.00)	9.00 (7.00)	-1.79	0.074	0.13	0.02 [0.00, 0.07]
F3 - impulsive sexual behaviors	3.00 (5.00)	2.00 (4.00)	-0.55	0.583	0.04	<0.01 [0.00, 0.03]
F4 - intentions to engage in risky sexual behaviors	0.00 (1.00)	0.00 (0.00)	-1.41	0.158	0.10	0.01 [0.00, 0.06]
F5 - risky anal intercourse	0.00 (3.00)	0.00 (2.75)	-0.37	0.709	0.03	<0.01 [0.00, 0.03]

Data are presented as median (IQR). The groups were compared using the two-sided Mann-Whitney U test. Z = standardized Mann-Whitney U test statistic; p = raw, unadjusted p-value; r = effect size calculated as |Z|/√N; η² = eta-squared effect size; CI = confidence interval.

### 3.3 Analysis of male respondents

#### 3.3.1 Type D personality and partner relationships in the male population

Partner relationship characteristics of the male cohort are presented in **Table 9**. Men with Type D personality reported a significantly lower lifetime number of sexual partners than those in the control group (Mann–Whitney U test; Z = -4.32; p < 0.001; effect size r = 0.40). Similar differences were observed in the number of sexual partners within the past 12 months (Z = -2.69; p = 0.007; effect size r = 0.25). Additionally, men with Type D personality reported engaging in sexual intercourse less frequently than control respondents at the unadjusted level (Z = -2.13; p = 0.033; effect size r = 0.20); however, this difference did not remain statistically significant after the Benjamini–Hochberg correction. No significant differences were observed between the groups with respect to relationship status or having a steady partner.

**Table 9:** Partner relationships and sexual activity characteristics in the studied group of men (N = 117).

A. Relationship and sexual activity characteristics						
Variable	Response	All	Type D personality		Control group	
Sexual orientation	Asexual	0.00%	0.00%		0.00%	
	Bisexual	6.80%	7.70%		6.20%	
	Heterosexual	76.90%	75.00%		78.50%	
	Homosexual	15.40%	17.30%		13.80%	
	Other	0.90%	0.00%		1.50%	
Regular sexual partner	Yes (one)	70.10%	73.10%		67.70%	
	Yes (many)	7.70%	3.80%		10.80%	
	No	22.20%	23.10%		21.50%	
Regular relationship	Yes, married	8.50%	5.80%		10.80%	
	Yes, partner	37.60%	36.50%		38.50%	
	No	53.80%	57.70%		50.80%	
Frequency of sexual intercourse	<1 time/month	19.10%	21.70%		17.20%	
	1–2 times/month	23.60%	28.30%		20.30%	
	1–2 times/week	32.70%	30.40%		34.40%	
	3–4 times/week	12.70%	10.90%		14.10%	
	>4 times/week	11.80%	8.70%		14.10%	
B. Group comparisons for sexual activity and satisfaction variables						
Variable	Control group Mdn (IQR)	Type D personality Mdn (IQR)	Z	p	r	η² [95% CI]
Frequency of sexual intercourse	3.00 (2.00)	2.00 (2.00)	-2.13	0.033*	0.2	0.04 [0.00, 0.13]
Number of sexual partners (lifetime)	5.00 (8.00)	2.00 (3.75)	-4.32	<0.001	0.4	0.16 [0.06, 0.29]
Number of sexual partners (last 12 months)	1.00 (1.75)	1.00 (0.00)	-2.69	0.007	0.25	0.06 [0.00, 0.17]
Sexual satisfaction (lifetime) (LS)	4.00 (1.00)	3.00 (1.00)	-4.47	<0.001	0.41	0.17 [0.06, 0.31]
Sexual satisfaction (last 12 months) (LS)	4.00 (2.00)	4.00 (1.00)	-1.88	0.06	0.17	0.03 [0.00, 0.12]

Part A presents categorical variables as percentages. Part B presents data as median (IQR). Group comparisons were performed using the two-sided Mann–Whitney U test. Mdn = median; IQR = interquartile range; Z = standardized Mann–Whitney U test statistic; p = raw, unadjusted p-value; r = effect size calculated as |Z|/√N; η² = eta-squared effect size; CI = confidence interval; LS = Likert scale. * This comparison was not statistically significant after the Benjamini–Hochberg correction. The frequency-of-intercourse variable was available for 110 men; percentages and the corresponding group comparison were based on valid responses.

#### 3.3.2 Type D personality, specific sexual activities, and age of initiation in the male population

**Table 10** presents a comparison of specific sexual activities in the male population. Respondents with Type D personality reported a significantly later age of initiation of oral sex (Z = −2.87; p = 0.004; r = 0.27) and vaginal sex (Z = −2.17; p = 0.030; r = 0.22). No significant differences were observed in the age of initiation for masturbation, petting, or anal sex. Furthermore, no significant differences were observed between the groups in overall engagement in various sexual activities.

**Table 10:** Sexual initiation and age at initiation of selected sexual activities among men (N = 117).

A. History of sexual initiation by type of activity							
Activity	Response	All	Type D personality				Control group
Masturbation	No	1.7%	1.9%				1.5%
	Yes	98.3%	98.1%				98.5%
Petting	No	8.5%	9.6%				7.7%
	Yes	91.5%	90.4%				92.3%
Oral sex	No	3.4%	5.8%				1.5%
	Yes	96.6%	94.2%				98.5%
Vaginal sex	No	21.4%	25.0%				18.5%
	Yes	78.6%	75.0%				81.5%
Anal sex	No	41.9%	40.4%				43.1%
	Yes	58.1%	59.6%				56.9%
B. Age at sexual initiation by type of activity							
Activity	Control group, Mdn (IQR)	Type D personality, Mdn (IQR)	Z	p	r	η² [95% CI]	
Masturbation	13.00 (2.00)	13.00 (2.00)	−0.15	0.880	0.01	<0.01 [0.00, 0.04]	
Petting	17.00 (3.50)	18.00 (4.00)	−1.95	0.051	0.19	0.04 [0.00, 0.13]	
Oral sex	17.00 (3.00)	19.00 (3.00)	−2.87	0.004	0.27	0.07 [0.01, 0.19]	
Vaginal sex	18.00 (3.00)	19.00 (3.00)	−2.17	0.030	0.22	0.05 [0.00, 0.14]	
Anal sex	18.00 (3.00)	19.00 (4.00)	−1.10	0.272	0.13	0.02 [0.00, 0.08]	

Part A presents percentages. Part B presents median (IQR). The groups were compared using the two-sided Mann–Whitney U test. Mdn = median; IQR = interquartile range; Z = standardized Mann–Whitney U test statistic; p = raw, unadjusted p-value; r = effect size calculated as |Z|/√N; η² = eta-squared effect size; CI = confidence interval.

#### 3.3.3 Type D personality and sexual life satisfaction in the male population

Lifetime sexual satisfaction was significantly lower among men with Type D personality (Mann–Whitney U test; Z = -4.47; p < 0.001; effect size r = 0.41). However, no significant differences were observed between the groups in sexual satisfaction over the past 12 months.

#### 3.3.4 Type D personality and self-reported sexual dysfunction symptoms in the male population

Specific symptoms of sexual dysfunction reported by respondents are presented in **Table 11**. No significant differences were observed between men with Type D personality and the control group in self-reported sexual dysfunction symptoms.

**Table 11:** Symptoms of sexual dysfunction in the studied group of men (N = 117).

Question	Sexual difficulty	Response	Control group, n (%)	Type D personality, n (%)	χ²	p	φ	OR [95% CI]
Have you noticed any of the following sexual difficulties during the past 12 months?	Decreased sexual desire	Yes	21 (32.3)	17 (33.3)	0.01	0.907	0.01	0.95 [0.44, 2.08]
		No	44 (67.7)	34 (66.7)				
	Erectile dysfunction	Yes	15 (23.1)	11 (21.6)	0.04	0.847	0.02	1.09 [0.45, 2.64]
		No	50 (76.9)	40 (78.4)				
	Dyspareunia	Yes	6 (9.4)	7 (13.7)	0.54	0.464	0.07	0.65 [0.20, 2.07]
		No	58 (90.6)	44 (86.3)				
	Premature ejaculation	Yes	14 (21.5)	17 (32.7)	1.85	0.174	0.13	0.56 [0.25, 1.29]
		No	51 (78.5)	35 (67.3)				
	Orgasmic disorder	Yes	23 (35.4)	16 (31.4)	0.21	0.650	0.04	1.20 [0.55, 2.61]
		No	42 (64.6)	35 (68.6)				
	Masturbatory anorgasmia	Yes	8 (12.3)	12 (23.1)	2.36	0.124	0.14	0.47 [0.17, 1.25]
		No	57 (87.7)	40 (76.9)				
During the past 12 months, have you noticed any of the following sexual difficulties in your partner?	Decreased sexual desire	Yes	18 (29.5)	16 (32.0)	0.08	0.777	0.03	0.89 [0.40, 2.00]
		No	43 (70.5)	34 (68.0)				
	Lubrication difficulties	Yes	8 (13.6)	8 (16.0)	0.13	0.720	0.03	0.82 [0.28, 2.38]
		No	51 (86.4)	42 (84.0)				
	Dyspareunia	Yes	23 (38.3)	17 (34.0)	0.22	0.638	0.04	1.21 [0.55, 2.64]
		No	37 (61.7)	33 (66.0)				
	Orgasmic disorder	Yes	15 (24.6)	16 (32.0)	0.75	0.387	0.08	0.69 [0.30, 1.59]
		No	46 (75.4)	34 (68.0)				
	Masturbatory anorgasmia	Yes	8 (13.3)	10 (20.8)	1.08	0.299	0.10	0.58 [0.21, 1.62]
		No	52 (86.7)	38 (79.2)				

Percentages were calculated using the number of valid responses for each item; denominators therefore vary across questions. Data are presented as n (%). χ² = chi-square test statistic; p = raw, unadjusted p-value; φ = phi coefficient (effect size); OR = odds ratio; CI = confidence interval. ORs compare the control group with the Type D personality group for the “Yes” response.

#### 3.3.5 Index of premature ejaculation and Type D personality in the male population

No statistically significant differences were observed between the Type D and control group in the Index of Premature Ejaculation (IPE) total score or its subscales. Detailed results are presented in **Table 12**.

**Table 12:** Premature-ejaculation-related outcomes in the studied group of men (N = 117).

IPE domain	Control group (n = 65), Mdn (IQR)	Type D personality (n = 52), Mdn (IQR)	Z	p	r	η² [95% CI]
IPE total score	19.00 (8.00)	22.00 (11.50)	−1.03	0.303	0.10	<0.01 [0.00, 0.07]
F1 – sexual satisfaction	4.00 (3.00)	5.00 (5.00)	−0.60	0.551	0.06	<0.01 [0.00, 0.06]
F2 – control	5.00 (4.00)	7.00 (6.00)	−1.11	0.269	0.10	0.01 [0.00, 0.08]
F3 – distress	9.00 (5.00)	8.00 (7.50)	−1.19	0.233	0.11	0.01 [0.00, 0.08]

Data are presented as median (IQR). The groups were compared using the two-sided Mann–Whitney U test. IPE = Index of Premature Ejaculation; Mdn = median; IQR = interquartile range; Z = standardized Mann–Whitney U test statistic; p = raw, unadjusted p-value; r = effect size calculated as |Z|/√N; η² = eta-squared effect size; CI = confidence interval.

#### 3.3.6 Risky sexual behaviors measured by the SRS in the male population

Men with Type D personality reported engaging in risky sexual behaviors significantly less frequently. This was reflected in a significantly lower total score on the Sexual Risk Survey (SRS) (Z = -4.07; p < 0.001; effect size r = 0.38) and lower scores in four out of five individual domains, as presented in **Table 13**.

**Table 13:** Sexual risk survey scores in the studied group of men (N = 117).

Variable	Control group (n = 65), Mdn (IQR)	Type D personality (n = 52), Mdn (IQR)	Z	p	r	η² [95% CI]
SRS total score	31.00 (29.00)	15.00 (15.50)	−4.07	<0.001	0.38	0.14 [0.04, 0.27]
F1 – sexual behaviors with casual partners	10.00 (16.50)	3.00 (6.75)	−3.54	<0.001	0.33	0.11 [0.02, 0.23]
F2 – risky sexual intercourse	9.00 (8.00)	5.00 (6.75)	−3.59	<0.001	0.33	0.11 [0.03, 0.23]
F3 – impulsive sexual behaviors	4.00 (8.00)	2.00 (3.00)	−3.31	<0.001	0.31	0.09 [0.02, 0.21]
F4 – intentions to engage in risky sexual behaviors	0.00 (4.00)	0.00 (1.00)	−2.51	0.012	0.23	0.05 [0.00, 0.16]
F5 – risky anal intercourse	1.00 (5.00)	0.00 (2.00)	−1.57	0.117	0.15	0.02 [0.00, 0.10]

Data are presented as median (IQR). The groups were compared using the two-sided Mann–Whitney U test. SRS = Sexual Risk Survey; Mdn = median; IQR = interquartile range; Z = standardized Mann–Whitney U test statistic; p = raw, unadjusted p-value; r = effect size calculated as |Z|/√N; η² = eta-squared effect size; CI = confidence interval.

### 3.4 Exploratory multivariable regression analyses

Prior to the regression analyses, model assumptions were assessed. For the FSFI, IPE, and SRS models, residual normality and homoscedasticity were considered met, and variance inflation factor values did not exceed 3, indicating no problematic multicollinearity. For the lifetime sexual satisfaction models, inference was based on HC3 robust standard errors, confidence intervals, t tests, and F tests. Influential observations were assessed using Cook’s distance; one highly influential observation was excluded from the SRS analysis.

#### 3.4.1 FSFI total score among women

The hierarchical regression analysis for the FSFI total score included 190 women who reported partnered sexual activity during the preceding four weeks. The covariate-only model was statistically significant, F (5, 184) = 2.45, p = 0.035, and had an adjusted R² of 0.037. Adding Type D personality in Step 2 significantly improved the model, ΔF (1, 183) = 11.09, p = 0.001, ΔR² = 0.054. The final model was statistically significant, F (6, 183) = 4.00, p < 0.001, and had an adjusted R² of 0.087. Stable relationship status was statistically significant in Step 1 but no longer remained significant after Type D personality was added. In the final model, Type D personality was the only statistically significant predictor and was associated with a lower FSFI total score (b = −2.39, 95% CI [−3.80, −0.97], β = −0.24, p = 0.001). Full results are presented in **Supplementary Table 1**.

#### 3.4.2 IPE total score among men

The covariate-only model for the IPE total score was not statistically significant, F (5, 111) = 1.61, p = 0.163, adjusted R² = 0.026. Adding Type D personality did not significantly improve the model, ΔF (1, 110) = 0.49, p = 0.486, ΔR² = 0.004. The final model was also not statistically significant, F (6, 110) = 1.42, p = 0.213, adjusted R² = 0.021. Type D personality was not associated with the IPE total score in the adjusted model (b = 1.38, 95% CI [−2.53, 5.29], β = 0.07, p = 0.486). Full results are presented in **Supplementary Table 2**.

#### 3.4.3 Lifetime sexual satisfaction

Separate hierarchical regression analyses were conducted for lifetime sexual satisfaction among women and men. Among women, the covariate-only model was statistically significant, F(5, 193) = 4.19, p = 0.001, adjusted R² = 0.085. Adding Type D personality significantly improved the model, ΔF (1, 192) = 7.26, p = 0.008, ΔR² = 0.030. In the final model, Type D personality was associated with lower lifetime sexual satisfaction (b = −0.36, 95% CI [−0.62, −0.10], β = −0.18, p = 0.008). Stable relationship status also remained statistically significant. The final model was statistically significant, F(6, 192) = 4.52, p < 0.001, and had an adjusted R² of 0.111. Full results are presented in **Supplementary Table 3**.

Among men, the covariate-only model was not statistically significant, F (5, 111) = 1.77, p = 0.126, adjusted R² = 0.040. Adding Type D personality resulted in a significant improvement, ΔF (1, 110) = 19.67, p < 0.001, ΔR² = 0.151. The final model was statistically significant, F (6, 110) = 5.18, p < 0.001, and had an adjusted R² of 0.190. Type D personality was associated with lower lifetime sexual satisfaction (b = −0.85, 95% CI [−1.23, −0.47], β = −0.39, p < 0.001). Stable relationship status also remained statistically significant. Full results are presented in **Supplementary Table 4**.

#### 3.4.4 SRS total score

The SRS regression analysis included 299 participants. Sixteen observations were not included because the frequency-of-intercourse variable could not be assigned to either of the two regression categories. In addition, one highly influential observation was excluded before the final analysis because its Cook’s distance exceeded 4. Variance inflation factor values were below 3, indicating no problematic multicollinearity. The covariate-only model was statistically significant, F (8, 290) = 14.93, p < 0.001, and had an adjusted R² of 0.272. Adding Type D personality in Step 2 significantly improved the model, ΔF (1, 289) = 4.87, p = 0.028, ΔR² = 0.012. The final model was statistically significant, F(9, 289) = 13.99, p < 0.001, with an adjusted R² of 0.282. In the final model, a higher lifetime number of sexual partners was associated with a higher SRS total score (b = 0.83, 95% CI [0.64, 1.02], β = 0.47, p < 0.001), whereas Type D personality was associated with a lower SRS total score (b = −3.81, 95% CI [−7.22, −0.41], β = −0.12, p = 0.028). Lifetime number of sexual partners was the strongest predictor in the final model. Full results are presented in **Supplementary Table 5**.

### 3.5 Magnitude and practical relevance of the observed effects

Among women, the statistically significant unadjusted associations were generally small. The association between Type D personality and meeting the FSFI cut-off was also small according to the phi coefficient, although the more than twofold higher odds may have potential clinical relevance and should be interpreted cautiously. In the adjusted models, adding Type D personality increased the explained variance by 5.4 percentage points for the FSFI total score, 3.0 percentage points for lifetime sexual satisfaction among women, 15.1 percentage points for lifetime sexual satisfaction among men, and 1.2 percentage points for the SRS total score. Its contribution to the IPE model was small and not statistically significant (ΔR² = 0.004). The standardized associations were modest for FSFI (β = −0.24), lifetime sexual satisfaction among women (β = −0.18), and SRS (β = −0.12). The standardized coefficient for lifetime sexual satisfaction was descriptively larger among men (β = −0.39) than women, although no formal between-sex comparison was performed. These findings indicate that statistical significance did not uniformly correspond to a large explanatory contribution.

## 4 Discussion

The present results indicate associations between Type D personality and several aspects of sexual life, although the observed pattern differed between women and men. Participants with Type D personality reported fewer lifetime sexual partners, and men reported later initiation of oral and vaginal sex. Men with Type D also showed a nominal difference in intercourse frequency, but this finding did not remain statistically significant after the Benjamini–Hochberg correction. These differences may be considered in the context of the social inhibition and avoidance characteristic of Type D personality, in line with previous research on avoidance, personality characteristics, and entry into romantic relationships (36–38). This interpretation remains tentative, as the present study did not assess the processes underlying sexual or relationship history. Lifetime sexual satisfaction was also lower in both women and men with Type D personality, consistent with reports of poorer sexual-domain quality of life in psoriasis and lower sexual life satisfaction in patients with non-cardiac chest pain (27, 39). These associations remained statistically significant after adjustment for the available demographic, relationship-related, and mental-health history covariates. The standardized association was descriptively larger among men than women, although this difference was not formally tested. Stable relationship status also remained associated with lifetime sexual satisfaction in both final models, suggesting that relationship context contributed alongside Type D personality. No corresponding differences were found for satisfaction during the previous 12 months. Lifetime and more recent ratings may capture partly different aspects of sexual experience or relationship context, but the present cross-sectional data do not allow these possibilities to be distinguished.

Type D personality was identified in 49.4% of the sample. This proportion is high, but not unprecedented in university populations. Previous studies reported rates of 39.2% among Japanese university students, 44% among Iranian students, and 53.3% among Polish physiotherapy students assessed during the COVID-19 pandemic (40–42). These estimates are not directly comparable because the studies differed in sample characteristics, cultural setting, and recruitment procedures. Recruitment through Facebook student groups may also have been associated with the participation of students who were particularly interested in psychological or sexual-health topics or who were experiencing greater distress. Selection bias is therefore a plausible contributor to the elevated proportion of participants meeting the Type D criteria, although its magnitude cannot be determined from the present data. The observed proportion should therefore be treated as a characteristic of this self-selected sample rather than an estimate for Polish university students in general.

Among the self-reported sexual difficulties, dyspareunia was more common in women with Type D personality. No corresponding differences were observed among men. Clinical studies have previously reported associations between Type D personality and poorer sexual functioning or sexual quality of life, while dyspareunia has been associated with psychological burden and reduced quality of life (26, 43). The absence of significant findings among men may reflect a lack of detectable association in this sample, but it may also be related to the limited sensitivity of single symptom-based questions.

The FSFI results provided a broader assessment of sexual functioning in women. Women with Type D personality had lower total scores and lower scores for arousal, lubrication, orgasm, and pain, whereas desire did not differ between the groups. They were also more than twice as likely to meet the FSFI cut-off for risk of clinically relevant sexual dysfunction. The adjusted analysis supported the association between Type D personality and poorer overall female sexual functioning. However, the final model explained only a limited proportion of variability in FSFI scores, consistent with the multifactorial nature of female sexual functioning. Most effect sizes were small, and the magnitude of these differences should not be overstated. Nevertheless, the cut-off result warrants further investigation in longitudinal and clinically characterized samples. From a practical perspective, the higher proportion of women meeting the FSFI cut-off suggests that Type D personality may help identify a subgroup reporting more sexual-function difficulties. However, the FSFI cut-off indicates risk rather than a clinical diagnosis, and the present findings do not support the use of Type D personality as a screening criterion. A previous study also reported an association between Type D personality and FSFI-defined risk of sexual dysfunction, while depressive symptoms and personality characteristics have been associated with orgasmic function and pain in young women (25, 44, 45). Negative emotionality, tension, or difficulties in communicating sexual needs may be relevant, although these factors were not measured in the present study.

No differences were found between men with and without Type D personality in the IPE total or domain scores. The adjusted analysis did not alter this conclusion, as Type D personality was not associated with the IPE total score after accounting for the available covariates and did not meaningfully improve the model. This result applies only to premature-ejaculation-related outcomes assessed with the IPE. The instrument focuses on ejaculatory control, satisfaction, and distress and is not directly comparable with the multidimensional FSFI. The present findings therefore should not be interpreted as evidence that Type D personality is unrelated to male sexual dysfunction more broadly. Previous studies have reported associations between Type D personality and erectile dysfunction and between Type D personality and higher premature-ejaculation symptom scores, although depressive symptoms and moderate-to-severe erectile dysfunction were the main independent predictors in the latter study (23, 24). Differences in the measured outcomes, psychological comorbidity, sample composition, and instrument choice should be considered when comparing the available findings.

Lower SRS scores were observed in both women and men with Type D personality, with stronger and more consistent differences among men. These results may be compatible with a more socially inhibited or avoidant interpersonal pattern, given broader associations of introversion with less risky sexual behavior and sensation seeking with greater risk-taking (46, 47). However, the present study did not assess the motives, opportunities, relationship circumstances, or decision-making processes underlying the reported behaviors. Lower SRS scores should therefore not be interpreted as evidence that Type D personality has a protective effect. In the adjusted regression model, Type D personality remained associated with a lower SRS total score, although its contribution to the model was modest, and lifetime number of sexual partners was the strongest predictor. These adjusted findings should be interpreted within the present sample because residual confounding cannot be excluded.

## 5 Limitations

This study has several limitations that should be considered when interpreting the findings. First, its cross-sectional design precludes conclusions regarding the directionality or causality of the associations between Type D personality and sexual functioning. Second, recruitment relied exclusively on voluntary participation through Facebook student groups, which may have introduced selection bias. Students who did not use Facebook, were not active in university-related groups, or were less willing to participate in a study addressing psychological and sexual-health topics may have been underrepresented. Conversely, students with a greater interest in these topics or experiencing greater psychological distress may have been more likely to participate. Because universities, Facebook groups, and individual participants were not randomly sampled, and no stratification or quota procedures were applied, the sample cannot be considered representative of Polish university students. The findings should therefore be generalized cautiously beyond students who could be reached through similar online recruitment channels. In addition, because IP addresses and other direct identifiers were not collected, duplicate participation could not be formally screened and cannot be entirely excluded. This self-selection may also have contributed to the relatively high proportion of participants meeting the DS14 criteria for Type D personality. No formal *a priori* sample size calculation was performed, which represents an important limitation of the study, particularly in relation to sex-stratified analyses. Although the *post-hoc* power analysis indicated acceptable power to detect medium effects in several analyses, the power of the male subgroup was slightly below the conventional threshold for continuous comparisons and limited for smaller categorical effects. Therefore, some non-significant findings in men, particularly those concerning IPE-assessed premature-ejaculation-related outcomes, may reflect a true lack of association, the specific measurement instrument used, or limited statistical power.

The study also relied on self-report measures. Validated instruments were used to assess Type D personality, female sexual functioning, premature-ejaculation-related outcomes, and risky sexual behaviors; however, some sociodemographic, relationship-related, sexual history, and self-reported dysfunction variables were assessed using an author-developed questionnaire. This questionnaire was reviewed by an expert in sexology and pre-tested among 30 medical university students for clarity, cultural appropriateness, acceptability, and technical functionality, but it was not formally psychometrically validated. Therefore, variables derived from this questionnaire should be interpreted as descriptive and exploratory. Male sexual functioning was not assessed with a comprehensive multidimensional instrument directly comparable to the FSFI. This asymmetry limits direct comparisons between women and men, and the male findings should therefore be interpreted as referring mainly to premature-ejaculation-related outcomes and selected self-reported dysfunction symptoms rather than to male sexual functioning as a whole. The IPE was administered in Polish using a Polish-language version reported in the original validation study as one of the available translations, but no separate peer-reviewed psychometric validation study of the Polish-language version was identified. Accordingly, IPE-based findings should be interpreted cautiously as exploratory premature-ejaculation-related outcomes. Self-report may also introduce recall and social-desirability bias and may be influenced by participants’ current emotional state.

Depressive and anxiety symptoms were not assessed using dedicated validated scales. Although self-reported history of depression and anxiety disorders was available and included as a covariate in the exploratory multivariable regression analyses, these historical binary variables do not capture the presence or severity of current symptoms. They therefore cannot fully distinguish the association of Type D personality, particularly its negative affectivity component, from the potential influence of current psychological distress. Residual confounding by depressive and anxiety symptoms cannot be excluded.

The multivariable regression analyses should be interpreted as exploratory and were limited to covariates available in the dataset. Although adjusted models were conducted for the primary continuous outcomes, they cannot isolate a causal effect of Type D personality. Several potentially important confounders, including medication use, chronic illness, substance use, relationship satisfaction, and history of sexual trauma or distressing sexual experiences, were not assessed in detail. The reported coefficients should therefore be interpreted as adjusted associations within the present sample rather than estimates of independent or causal effects.

Future longitudinal studies should use comprehensive gender-specific measures of sexual functioning, validated mental-health symptom scales, and broader adjustment for potential confounders.

## 6 Conclusions

1. In this exploratory cross-sectional study of Polish university students, Type D personality was associated with selected indicators of sexual functioning, sexual satisfaction, and sexual behavior. The findings do not establish causality, directionality, or underlying mechanisms.
2. Among women, Type D personality remained associated with a lower FSFI total score after adjustment for the available covariates. Unadjusted analyses additionally showed lower arousal, lubrication, orgasm, and pain scores and higher odds of meeting the FSFI cut-off for risk of clinically relevant sexual dysfunction.
3. Type D personality remained associated with lower lifetime sexual satisfaction after adjustment in both women and men. Type D personality was not associated with the IPE total score after adjustment, and the male findings should not be generalized beyond premature-ejaculation-related outcomes.
4. Type D personality remained modestly associated with a lower SRS total score after adjustment for the available covariates, although its incremental explanatory contribution was small. Lifetime number of sexual partners was the strongest predictor of the SRS total score.
5. The results require confirmation in longitudinal and clinically characterized studies using validated mental-health symptom measures and broader control of potential confounders before implications for screening or clinical practice can be established.

## Data Availability

The raw data supporting the conclusions of this article will be made available by the authors, without undue reservation.
