# Supplementary material for: Type D personality, sexual functioning, sexual satisfaction, and risky sexual behaviors among Polish university students: a cross-sectional study
Source: Front Psychiatry. 2026 Sep 10;17:1866935. doi: 10.3389/fpsyt.2026.1866935 (PMC13601216; doi:10.3389/fpsyt.2026.1866935)
Supplement: Supplementary file 1 [file DataSheet1.pdf]

Supplementary Table S1. Hierarchical linear regression analysis predicting FSFI total score among women

A. Model summary

Model	F (df1, df2)	p	Adjusted R^2	ΔF (df1, df2)	p for ΔF	ΔR^2
**1**	2.45 (5, 184)	0.035	0.037	—	—	—
**2**	4.00 (6, 183)	<0.001	0.087	11.09 (1, 183)	0.001	0.054

B. Regression coefficients

Predictor	b [95% CI]	SE	β	t	p
**Model 1**					
Constant	30.09 [21.97, 38.21]	4.12	—	7.31	<0.001
Age	0.08 [−0.07, 0.23]	0.08	0.10	1.05	0.296
Stable relationship	−1.45 [−2.89, −0.01]	0.73	−0.15	−1.98	0.049
Self-reported history of depression	0.02 [−2.08, 2.11]	1.06	0.00	0.01	0.988
Self-reported history of anxiety disorder	−1.55 [−3.61, 0.51]	1.05	−0.13	−1.48	0.139
Having children	−1.02 [−3.78, 1.75]	1.40	−0.07	−0.72	0.470
**Model 2**					
Constant	31.34 [23.40, 39.28]	4.02	—	7.79	<0.001
Age	0.05 [−0.10, 0.19]	0.07	0.06	0.61	0.541
Stable relationship	−1.31 [−2.72, 0.09]	0.71	−0.13	−1.84	0.067
Self-reported history of depression	0.52 [−1.55, 2.58]	1.04	0.04	0.49	0.622
Self-reported history of anxiety disorder	−1.42 [−3.43, 0.59]	1.02	−0.12	−1.40	0.164
Having children	−0.72 [−3.41, 1.98]	1.37	−0.05	−0.52	0.601
Type D personality	−2.39 [−3.80, −0.97]	0.72	−0.24	−3.33	0.001

Note. N = 190. Dependent variable: FSFI total score. Model 1 included age, stable relationship status, self-reported history of depression, self-reported history of anxiety disorder, and having children; Model 2 additionally included Type D personality. Coding: stable relationship, 1 = yes and 2 = no; having children, 1 = yes and 2 = no; self-reported history of depression and anxiety disorder, 1 = yes and 0 = no; Type D personality, 0 = control and 1 = Type D. b = unstandardized regression coefficient; CI = confidence interval; SE = standard error; β = standardized regression coefficient; t = Student's t statistic; F = F statistic; ΔF = change in F; adjusted R^2 = adjusted coefficient of determination; ΔR^2 = change in unadjusted R^2. FSFI analyses were restricted to women who reported partnered sexual activity during the preceding four weeks.

Supplementary Table S2. Hierarchical linear regression analysis predicting IPE total score among men

A. Model summary

Model	F (df1, df2)	p	Adjusted R^2	ΔF (df1, df2)	p for ΔF	ΔR^2
**1**	1.61 (5, 111)	0.163	0.026	—	—	—
**2**	1.42 (6, 110)	0.213	0.021	0.49 (1, 110)	0.486	0.004

B. Regression coefficients

Predictor	b [95% CI]	SE	β	t	p
**Model 1**					
Constant	35.35 [12.67, 58.03]	11.45	—	3.09	0.003
Age	−0.08 [−0.53, 0.37]	0.23	−0.04	−0.35	0.725
Stable relationship	−4.75 [−8.70, −0.79]	2.00	−0.23	−2.38	0.019
Self-reported history of depression	−2.40 [−9.32, 4.52]	3.49	−0.08	−0.69	0.493
Self-reported history of anxiety disorder	0.78 [−5.60, 7.17]	3.22	0.03	0.24	0.809
Having children	−3.67 [−10.62, 3.28]	3.51	−0.11	−1.05	0.298
**Model 2**					
Constant	35.05 [12.30, 57.80]	11.48	—	3.05	0.003
Age	−0.08 [−0.53, 0.38]	0.23	−0.04	−0.33	0.739
Stable relationship	−4.82 [−8.79, −0.85]	2.00	−0.23	−2.41	0.018
Self-reported history of depression	−2.38 [−9.31, 4.56]	3.50	−0.08	−0.68	0.499
Self-reported history of anxiety disorder	0.51 [−5.94, 6.95]	3.25	0.02	0.16	0.877
Having children	−3.81 [−10.78, 3.17]	3.52	−0.11	−1.08	0.282
Type D personality	1.38 [−2.53, 5.29]	1.97	0.07	0.70	0.486

Note. N = 117. Dependent variable: IPE total score. Model 1 included age, stable relationship status, self-reported history of depression, self-reported history of anxiety disorder, and having children; Model 2 additionally included Type D personality. Coding: stable relationship, 1 = yes and 2 = no; having children, 1 = yes and 2 = no; self-reported history of depression and anxiety disorder, 1 = yes and 0 = no; Type D personality, 0 = control and 1 = Type D. b = unstandardized regression coefficient; CI = confidence interval; SE = standard error; β = standardized regression coefficient; t = Student's t statistic; F = F statistic; ΔF = change in F; adjusted R^2 = adjusted coefficient of determination; ΔR^2 = change in unadjusted R^2. IPE = Index of Premature Ejaculation.

Supplementary Table S3. Hierarchical linear regression analysis predicting lifetime sexual satisfaction among women

A. Model summary

Model	F (df1, df2)	p	Adjusted R²	ΔF (df1, df2)	p for ΔF	ΔR²
**1**	4.19 (5, 193)	0.001	0.085	—	—	—
**2**	4.52 (6, 192)	<0.001	0.111	7.26 (1, 192)	0.008	0.030

B. Regression coefficients

Predictor	b [95% CI]	SE	β	t	p
**Model 1**					
Constant	4.81 [3.52, 6.11]	0.66	—	7.33	<0.001
Age	0.01 [−0.02, 0.03]	0.01	0.06	0.73	0.466
Stable relationship	−0.43 [−0.71, −0.15]	0.14	−0.21	−3.03	0.003
Self-reported history of depression	−0.23 [−0.60, 0.15]	0.19	−0.09	−1.18	0.238
Self-reported history of anxiety disorder	−0.35 [−0.73, 0.04]	0.20	−0.14	−1.77	0.078
Having children	−0.26 [−0.69, 0.16]	0.21	−0.09	−1.23	0.220
**Model 2**					
Constant	4.97 [3.61, 6.32]	0.69	—	7.23	<0.001
Age	0.01 [−0.02, 0.03]	0.01	0.03	0.37	0.712
Stable relationship	−0.41 [−0.69, −0.13]	0.14	−0.21	−2.90	0.004
Self-reported history of depression	−0.16 [−0.54, 0.23]	0.20	−0.06	−0.80	0.423
Self-reported history of anxiety disorder	−0.33 [−0.72, 0.06]	0.20	−0.13	−1.68	0.095
Having children	−0.21 [−0.63, 0.22]	0.22	−0.07	−0.97	0.332
Type D personality	−0.36 [−0.62, −0.10]	0.13	−0.18	−2.70	0.008

Note. N = 199. Dependent variable: lifetime sexual satisfaction, assessed on a 1–5 Likert scale, with higher scores indicating greater satisfaction. Model 1 included age, stable relationship status, self-reported history of depression, self-reported history of anxiety disorder, and having children; Model 2 additionally included Type D personality. Coding: stable relationship, 1 = yes and 2 = no; having children, 1 = yes and 2 = no; self-reported history of depression and anxiety disorder, 1 = yes and 0 = no; Type D personality, 0 = control and 1 = Type D. b = unstandardized regression coefficient; CI = confidence interval; SE = standard error; β = standardized regression coefficient; t = Student's t statistic; F = F statistic; ΔF = change in F; adjusted R^2 = adjusted coefficient of determination; ΔR^2 = change in unadjusted R^2. HC3 robust standard errors, 95% confidence intervals, t statistics, p-values, and F tests are reported; adjusted R^2 and ΔR^2 are based on the underlying ordinary least-squares models. ΔF represents the robust one-degree-of-freedom test for the addition of Type D personality.

Supplementary Table S4. Hierarchical linear regression analysis predicting lifetime sexual satisfaction among men

A. Model summary

**Model**	**F (df1, df2)**	**p**	**Adjusted R^2**	**ΔF (df1, df2)**	**p for ΔF**	**ΔR^2**
**1**	1.77 (5, 111)	0.126	0.040	—	—	—
**2**	5.18 (6, 110)	<0.001	0.190	19.67 (1, 110)	<0.001	0.151

B. Regression coefficients

**Predictor**	**b [95% CI]**	**SE**	**β**	**t**	**p**
**Model 1**					
Constant	5.75 [2.96, 8.54]	1.41	—	4.09	<0.001
Age	−0.01 [−0.07, 0.04]	0.03	−0.06	−0.46	0.644
Stable relationship	−0.54 [−0.95, −0.13]	0.21	−0.25	−2.59	0.011
Self-reported history of depression	0.14 [−0.40, 0.68]	0.27	0.04	0.52	0.607
Self-reported history of anxiety disorder	−0.02 [−0.56, 0.52]	0.27	−0.01	−0.09	0.932
Having children	−0.43 [−1.24, 0.37]	0.41	−0.13	−1.07	0.288
**Model 2**					
Constant	5.94 [3.13, 8.74]	1.42	—	4.19	<0.001
Age	−0.01 [−0.07, 0.04]	0.03	−0.07	−0.57	0.573
Stable relationship	−0.49 [−0.89, −0.10]	0.20	−0.23	−2.46	0.015
Self-reported history of depression	0.13 [−0.49, 0.74]	0.31	0.04	0.41	0.685
Self-reported history of anxiety disorder	0.15 [−0.48, 0.77]	0.32	0.05	0.47	0.638
Having children	−0.35 [−1.12, 0.43]	0.39	−0.10	−0.89	0.375
Type D personality	−0.85 [−1.23, −0.47]	0.19	−0.39	−4.44	<0.001

Note. N = 117. Dependent variable: lifetime sexual satisfaction, assessed on a 1–5 Likert scale, with higher scores indicating greater satisfaction. Model 1 included age, stable relationship status, self-reported history of depression, self-reported history of anxiety disorder, and having children; Model 2 additionally included Type D personality. Coding: stable relationship, 1 = yes and 2 = no; having children, 1 = yes and 2 = no; self-reported history of depression and anxiety disorder, 1 = yes and 0 = no; Type D personality, 0 = control and 1 = Type D. b = unstandardized regression coefficient; CI = confidence interval; SE = standard error; β = standardized regression coefficient; t = Student's t statistic; F = F statistic; ΔF = change in F; adjusted R^2 = adjusted coefficient of determination; ΔR^2 = change in unadjusted R^2. HC3 robust standard errors, 95% confidence intervals, t statistics, p-values, and F tests are reported; adjusted R^2 and ΔR^2 are based on the underlying ordinary least-squares models. ΔF represents the robust one-degree-of-freedom test for the addition of Type D personality.

Supplementary Table S5. Hierarchical linear regression analysis predicting SRS total score

A. Model summary

Model	F (df1, df2)	p	Adjusted R^2	ΔF (df1, df2)	p for ΔF	ΔR^2
**1**	14.93 (8, 290)	<0.001	0.272	—	—	—
**2**	13.99 (9, 289)	<0.001	0.282	4.87 (1, 289)	0.028	0.012

B. Regression coefficients

Predictor	b [95% CI]	SE	β	t	p
**Model 1**					
Constant	20.59 [−1.02, 42.21]	10.98	—	1.87	0.062
Sex	0.50 [−2.90, 3.90]	1.73	0.02	0.29	0.773
Age	0.11 [−0.24, 0.45]	0.18	0.04	0.63	0.531
Lifetime number of sexual partners	0.86 [0.68, 1.05]	0.09	0.49	9.16	<0.001
Lifetime sexual satisfaction	0.78 [−0.91, 2.47]	0.86	0.05	0.91	0.363
Having children	−5.05 [−11.09, 1.00]	3.07	−0.10	−1.64	0.101
Frequency of sexual intercourse	0.57 [−3.78, 4.92]	2.21	0.01	0.26	0.796
Self-reported history of depression	1.57 [−3.60, 6.74]	2.63	0.04	0.60	0.551
Self-reported history of anxiety disorder	−0.87 [−5.94, 4.20]	2.58	−0.02	−0.34	0.736
**Model 2**					
Constant	24.26 [2.54, 45.99]	11.04	—	2.20	0.029
Sex	0.28 [−3.11, 3.66]	1.72	0.01	0.16	0.872
Age	0.09 [−0.25, 0.44]	0.17	0.03	0.53	0.595
Lifetime number of sexual partners	0.83 [0.64, 1.02]	0.10	0.47	8.72	<0.001
Lifetime sexual satisfaction	0.29 [−1.45, 2.02]	0.88	0.02	0.32	0.747
Having children	−4.69 [−10.71, 1.32]	3.06	−0.10	−1.53	0.126
Frequency of sexual intercourse	0.69 [−3.63, 5.01]	2.20	0.02	0.31	0.753
Self-reported history of depression	2.18 [−2.98, 7.35]	2.63	0.05	0.83	0.406
Self-reported history of anxiety disorder	−0.71 [−5.75, 4.33]	2.56	−0.02	−0.28	0.781
Type D personality	−3.81 [−7.22, −0.41]	1.73	−0.12	−2.21	0.028

Note. N = 299. Dependent variable: SRS total score. The regression was based on complete cases. Sixteen participants had no usable value for the dichotomized frequency-of-intercourse variable, and one highly influential observation was excluded based on Cook's distance. Model 1 included sex, age, lifetime number of sexual partners, lifetime sexual satisfaction, having children, frequency of sexual intercourse, and self-reported history of depression and anxiety disorder; Model 2 additionally included Type D personality. Coding: sex, 1 = women, 2 = men; having children, 1 = yes, 2 = no; frequency of sexual intercourse, 1 = less than once per month, 2 = more often than once per month; depression and anxiety disorder, 1 = yes, 0 = no; Type D personality, 0 = control, 1 = Type D. b = unstandardized coefficient; CI = confidence interval; SE = standard error; β = standardized coefficient; t = Student's t statistic; F = F statistic; ΔF = change in F; adjusted R^2 = adjusted coefficient of determination; ΔR^2 = change in unadjusted R^2.
